# The global distribution of CCR5 delta 32 polymorphism: role in HIV-1 protection

**DOI:** 10.1186/1471-2334-12-S1-O16

**Published:** 2012-05-04

**Authors:** Anuroopa Gupta, Harish Padh

**Affiliations:** 1 Department of Cellular and Molecular Biology, B. V. Patel Pharmaceutical Education & Research Development (PERD) Centre, Ahmedabad, India

## Background

The human immunodeficiency virus type 1 (HIV-1) infection occurs by binding to CD4+ receptor and chemokine receptor 5 (CCR5) or the CXC chemokine receptor (CXCR4). A mutation in the CCR5 gene, 32 base pair deletion consequences a truncated protein that is not expressed on the cell surface. The deletion confers resistance to HIV-1 infection and slows the progression of AIDS in HIV infected individuals. The worldwide distribution of CCR5delta 32 polymorphism was congregated by retrieving the data from literature and genotyping new population samples. A comprehensive resource of frequency data for CCR5delta 32 polymorphism in different population samples was created.

## Methods

The data for different populations was obtained from literature. In order to investigate the genetic variation in CCR5 gene in new population, we analyzed 257 healthy control individuals. We examined the CCR5 32 base pair deletion (CCR5-Δ32) by conventional polymerase chain reaction (PCR).

## Results

The genotype frequency distribution of CCR5 in new population was found to be (CCR5 / CCR5: 98%, CCR5 / CCR5-Δ32: 2% and CCR5-Δ32 / CCR5-Δ32: 0%) in healthy control.

## Conclusion

The allele frequency of CCR5-Δ32 observed for new population is 1% which is compared with the other populations. The polymorphism CCR5-Δ32 is primarily found in European population. Compared to our data, frequency of delta 32 deletion is observed at high frequency in European populations. Our data of delta 32 deletion is significantly different from Caucasians (p<0.00000001), Africans (p<0.01458) and Europeans (p< 0.00000001).

